# First person – Md Tangigul Haque

**DOI:** 10.1242/bio.062163

**Published:** 2025-08-06

## Abstract

First Person is a series of interviews with the first authors of a selection of papers published in Biology Open, helping researchers promote themselves alongside their papers. Md Tangigul Haque is first author on ‘ Heatwaves reduce mating frequency in an aquatic insect’, published in BiO. Md Tangigul is a PhD candidate in the lab of Md Kawsar Khan at Macquarie University, Sydney, Australia, investigating the impact of climate change on insect physiology, morphology, and behaviour, with a focus on thermal tolerance.


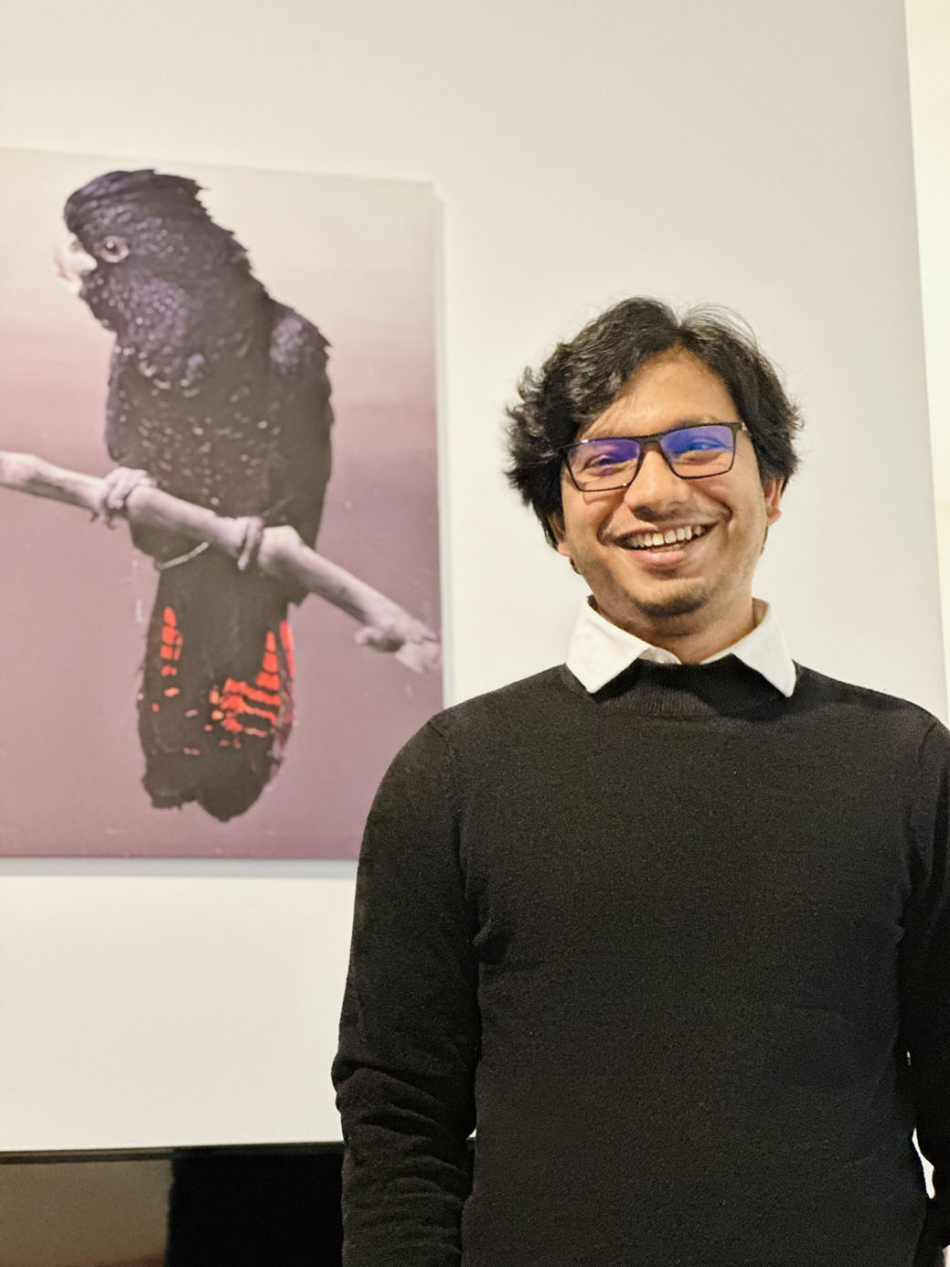


**Md Tangigul Haque**

**Describe your scientific journey and your current research focus**

My scientific journey began during my undergraduate studies in biochemistry and molecular biology at Shahjalal University of Science and Technology in Bangladesh. Over time, I developed an interest in field-based and laboratory-based research. Currently, my research focuses on investigating the impact of climatic factors on organisms' physiology, morphology, and behaviour. In addition, I am interested in understanding how organisms adapt to changing climates through various strategies, including the role of gut microbiomes.

**Who or what inspired you to become a scientist?**

Honestly, there are many things that inspired me to become a scientist. My curiosity about how organisms function began in high school and gradually deepened through hands-on-experiences in both school and university laboratories. I am very eager to explore the unknown, regardless of the setting (be it in a lab or in the field) or the organism involved. In addition, I have been greatly inspired by my undergraduate and graduate supervisors, as well as my colleagues, who have continuously encouraged and supported my scientific journey.

**How would you explain the main finding of your paper?**

We focused on understanding the impact of heatwaves in a natural setting using the damselfly as a model system. Our study revealed that natural heatwaves significantly reduce mating frequency in *Xanthagrion erythroneurum* damselflies, while showing no change in flight activity and local abundance. However, if heatwaves or other extreme events continue to increase, there may be long-term implications for population viability.

**What are the potential implications of this finding for your field of research?**

Our study revealed that understanding the impact of heatwaves in natural settings is more complex and challenging, albeit of great significance, alongside controlled laboratory conditions. Species may show behaviour changes or provide behavioural signs before exhibiting long-term serious consequences. These findings highlight the importance of monitoring and incorporating behavioural assessments into ecological monitoring and species risk assessment, which are ultimately necessary for species conservation and biodiversity management.

**Figure:**
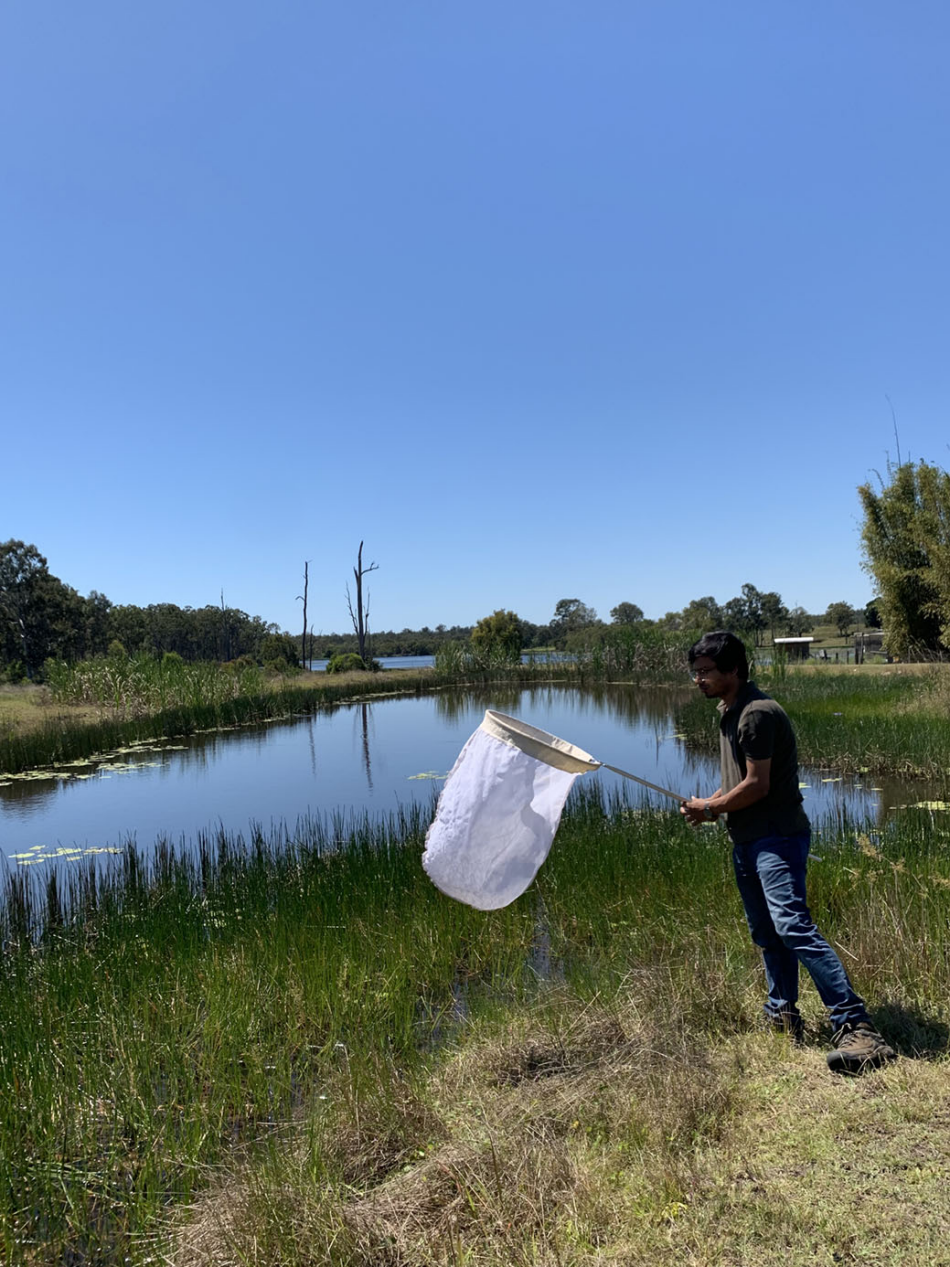
Collecting samples for my research work.

**Which part of this research project was the most rewarding?**

The most rewarding part of this research project was that, for the first time, our main protocol worked successfully in a natural setting. We trialled the protocol several times before the actual heatwaves, and it worked effectively. This was the most challenging part of this research project, as conducting three to four hours of fieldwork during heatwaves was not an easy task. One of our main challenges was that we might only get few heatwave days this year, so the protocol needed to work. I felt so accomplished when we successfully completed the project.

**What do you enjoy most about being an early-career researcher?**

As an early-career researcher, I believe I have the opportunity to learn every day and explore new ideas continuously. It always provides new challenges, which I feel so accomplished when I am able to overcome. In addition, it provides me with an opportunity to grow nationally and internationally through presenting my research at various conferences.

**What piece of advice would you give to the next generation of researchers?**

My key advice to the next generation of researchers is to stay committed to research, rather than focusing on specific outcomes. All results, including both positive and negative, are valuable contributions to scientific understanding. Sometimes, there will be hurdles, but believe in yourself, and face challenges with confidence. Additionally, aligning your research with your interests may help you stay engaged and motivated over a long period of time. Be patient – success will be yours.

Be patient – success will be yours

**What's next for you?**

My first priority is to successfully submit my PhD thesis. Following that, I aim to secure a postdoctoral research or research assistant position to contribute more to the scientific community.
